# Incidence density and factors associated with peripheral neuropathy among women with breast cancer during taxane-based chemotherapy

**DOI:** 10.1038/s41598-022-14870-y

**Published:** 2022-06-23

**Authors:** Nida Rattanakrong, Akkradate Siriphorn, Sujitra Boonyong

**Affiliations:** 1 Human Movement Performance Enhancement Research Unit, Department of Physical Therapy, Faculty of Allied Health Sciences, Chulalongkorn University, 154 Rama I Road, Wangmai, Pathumwan, Bangkok, Thailand; 2 Department of Rehabilitation Medicine, Physical Therapy Unit, Chulabhorn Hospital, Bangkok, Thailand

**Keywords:** Breast cancer, Cancer, Neurology, Neurological disorders, Peripheral neuropathies

## Abstract

This work aimed to determine the incidence density of taxane-induced peripheral neuropathy (TIPN) and its risk factors among women with breast cancer. One hundred and forty-one women with breast cancer participated in this cohort study. TIPN symptoms were evaluated with the European Organization for Research and Treatment of Cancer CIPN specific self-report questionnaire (EORTC QOL-CIPN20) at five-time points throughout chemotherapy treatment. Over three months, 125 (89%) and 59 (44.03%) women with breast cancer were identified with sensory and motor neuropathy, respectively. The sensory neuropathy incidence density was 21 per 1000 person-days. The motor neuropathy incidence density was 6 per 1000 person-days. This study discovered a significant link between age and the incidence density of sensory neuropathy (HR = 1.02; 95% CI: 1.01–1.05) as well as motor neuropathy (HR = 1.05; 95% CI: 1.01–1.08). These findings imply that screening may be necessary to detect early TIPN symptoms and provide appropriate rehabilitation programs, particularly for elderly persons.

## Introduction

A frequent consequence linked to taxane-based chemotherapy is taxane-induced peripheral neuropathy (TIPN), which presents with sensory and motor neuropathy symptoms. The TIPN symptom mechanism is not entirely understood. Changes in the structure of the axonal microtubule and mitochondria have been proposed, leading to deficiencies in axonic energy delivery^1^. Several studies have suggested that the development of TIPN could be associated with functional decline, increased risk of falls, and diminished quality of life (QOL)^2–4^. Unfortunately, no drugs exist for the prevention or effective treatment of TIPN^5^. The possible risk factors of TIPN involve patient characteristics (age, BMI, amount of medical comorbidity), the chemical dose and schedules (cumulative dose, dose intensity, weekly or tri-weekly), and the administration of multiple neurotoxic agents^1,6–8^.

TIPN-involved sensory neuropathy may appear as bilateral symptoms in the tips of the toes or fingers, such as numbness, tingling, and neuropathic pain^6^. Motor neuropathy symptoms, which involve distal muscular weakness, muscle cramps, and a dropping foot, are typically less frequent than sensory neuropathy. In addition, motor neuropathy seems to increase the risk of falls. Previous studies have shown a greater risk of falls in patients with motor neuropathy symptoms compared to those with sensory neuropathy symptoms^9^. Therefore, information regarding how many patients develop sensory and motor neuropathy is essential to minimize the consequences of TIPN**.**

The most common form of cancer found among women is breast cancer, which is typically treated with taxane-based chemotherapy^1^. Previous studies have reported the cumulative incidence and prevalence of TIPN symptoms in women with breast cancer who received taxane-based chemotherapy. A high prevalence for all grades of TIPN symptoms has been reported, ranging from 57 to 83%, with 2 to 33% developing severe neuropathy^4,6,10^. However, a lack of evidence remains concerning the incidence density of TIPN symptoms, particularly concerning sensory and motor neuropathy, in women with breast cancer. Although several studies have reported the prevalence and incidence of TIPN symptoms, no studies have reported the incidence density^4,6,10^. The prevalence indicates the number of pre-existing and new cases of disease in the population at a certain time, whereas the incidence is limited to new cases. Two commonly used types of incidences are cumulative incidence and incidence density. The cumulative incidence does not account for dropouts and time at risk, indicating that it offers an estimate of the risk of acquiring disease rather than the rate. On the other hand, the incidence density is calculated by dividing the number of new cases during study follow-up by the total person-days at risk. The days of observation have been calculated from the day of enrollment to the date of disease development, loss of follow-up, death, or trial completion^11^. In addition, the risk factors related to incidence density have not been investigated. Therefore, this study aimed to determine the incidence density of sensory and motor neuropathy symptoms as well as to examine the potential risk factors among women with breast cancer undergoing taxane-based chemotherapy.

## Materials and methods

### Design and setting

This prospective cohort study was performed to determine the incidence density of sensory and motor neuropathy as well as to examine the association between possible factors and TIPN incidence density among women with breast cancer during taxane-based chemotherapy. Patients were recruited from the National Cancer Institute of Thailand, King Chulalongkorn Memorial Hospital, and Bhumibol Adulyadej Hospital, Thailand, between October 2020 and July 2021.

### Participants

One hundred and forty-one women with breast cancer participated in this cohort study. This research related to human use has complied with all relevant national regulations and institutional policies and has followed the tenets of the Declaration of Helsinki. The research was approved by the Ethics Review Committee for Research Involving Human Projects, Chulalongkorn University (COA No. 209/2020), National Cancer Institute of Thailand (COA No. 025/2020), King Chulalongkorn Memorial Hospital (COA No. 001/2021), and Bhumibol Adulyadej Hospital (under study ID: 13/63) before data collection.

The eligibility criteria were (1) age 35–65 years, (2) plan to be treated with taxane-based chemotherapy, and (3) ability to communicate and understand the Thai language. Patients were excluded if they (1) had musculoskeletal diseases or neurological conditions with peripheral neuropathic signs or (2) had received other chemotherapy agents.

### Procedures

Before participating in this study, each participant was informed about the purpose of the study and testing procedures, and written informed consent was obtained. The participants were asked to report the number and type of medical comorbidities and the total number of types of medications usually taken. Participants were then allocated into two subgroups for further analysis: those aged equal to or older than 60 years and those under 60 years.

TIPN symptoms and severity were evaluated by the European Organization for Research and Treatment of Cancer CIPN specific self-report questionnaire (EORTC QOL-CIPN20; Thai version) at five time points: (1) before the initiation of baseline chemotherapy, (2–4) before the start of subsequent chemotherapy cycles, and (5) within 30 days after the last cycle of taxane-based chemotherapy was received. The EORTC QLQ-CIPN20 (Thai version) comprises 20 items divided into three subscales assessing sensory, motor, and autonomic symptoms. Each item is scored on a 4-point Likert scale ranging from 1 to 4 (1 = “not at all,” 2 = “a little,” 3 = “quite a bit,” 4 = “very much”)^12^. The EORTC QLQ-CIPN20 was already translated into Thai with good internal consistency (Cronbach’s α = 0.79)^13^. The psychometric properties of the EORTC QLQ-CIPN20 (Thai version) were completed before data collection. Excellent test–retest reliability (ICC_3,1_ = 0.84–0.95) and excellent inter-rather reliability (ICC_2,1_ = 0.78–0.94) were established. To identify sensory neuropathy, four items concerning finger and toe numbness or tingling were used. If the participants indicated severity at any level including a little, quite a bit, or very much in any category, they were identified as having sensory neuropathy symptoms^14,15^. In addition, four items related to difficulty in use or weakness of the hand or leg were used to identify motor neuropathy. If the participants indicated severity at any level including a little, quite a bit, or very much in any category, they were identified as having motor neuropathy symptoms.

### Statistical analysis

Data were analyzed using SPSS Statistics version 23 for Windows (IBM, Armonk, NY, USA). Descriptive statistical analysis was used for baseline data and severity of TIPN symptoms. In terms of TIPN incidence density, a chance for initial sensory and motor neuropathy symptoms occurring during the period of taxane-based chemotherapy. The incidence density formula is given as the number of initial TIPN symptoms divided by the total person-days at risk. The person-days are an estimate of the actual days at risk of developing TIPN symptoms. The researchers totaled the days of observation from participant registration to the date of first TIPN symptoms, termination, last follow-up, and death. The Cox proportional-hazards model was used to determine the association between the relevant covariates and the initial TIPN symptoms and then reported as an unadjusted and adjusted hazard ratio (HR) with a 95% confidence interval (CI). Univariate analysis was used to describe the relationship between time to present the initial sensory and motor neuropathy symptoms and relevant covariates factors, including patient characteristics (age and BMI), the chemotherapy conditions (line of therapy, regimen, number of cycles received, and cumulative dose), and health and medical conditions (number and type of medical comorbidities, and number of tablets usually taken). The covariates factors with a p-value smaller than 0.25 were considered the retaining variables and used for the adjustment in the multivariate analysis^16,17^. Age, the number of cycles received, dyslipidemia, and diabetes were revealed as retaining variables for initial sensory neuropathy symptoms. Age, cumulative dose, hypertension, dyslipidemia, diabetes, other medication comorbidities, number of medication comorbidities, and number of tablets usually taken were revealed as retaining variables for initial motor neuropathy symptoms. Next, the multivariable regression model was performed with a backward stepwise approach, as well as the retaining variables. The incidence densities for sensory and motor neuropathy symptoms were evaluated by the Kaplan–Meier (KM) estimate and illustrated as a KM curve. The log-rank test was performed to examine the survival functions of sensory and motor neuropathy symptoms among participant subgroups based on potential risk factors. The significance level was set at *p* < 0.05.

## Results

### Sample demographic characteristics

One hundred and forty-one women with breast cancer participated in this cohort study. The demographic characteristics of the participants are shown in Table 1. At baseline, the average age and BMI were 50.65 years (SD = 8.75) and 25.62 kg/m^2^ (SD = 12.24). The most common medical comorbidities were hypertension (26.95%), dyslipidemia (17.73%), and diabetes (9.20%). Although none of the participants experienced sensory neuropathy, 7 (4.96%) experienced motor neuropathy at the beginning. At the end of the study, most of the participants received treatment in the form of adjuvant chemotherapy with regimens of taxane-based chemotherapy including paclitaxel 175 mg/m^2^ given every 3 weeks for four cycles. The average cumulative dosage was 1131.63 mg (SD = 268.75). The number of medical comorbidities and total number of medication types usually taken is also reported in Table 1.

**Table 1:** Demographic and clinical characteristics of the participants.

Characteristic (n = 141)	n (%)
**Baseline**	
Age (yrs.), mean (SD)	50.65 (8.75)
Weight (kg), mean (SD)	60.17 (11.68)
Height (cm), mean (SD)	155.32 (9.65)
BMI (kg/m^2^), mean (SD)	25.62 (12.24)
Medical comorbidities	
Hypertension	38 (26.95)
Dyslipidemia	25 (17.73)
Diabetes	13 (9.20)
Hepatitis	4 (2.84)
Gerd	3 (2.13)
Allergy	3 (2.13)
Other	11 (7.80)
Number of medical comorbidities	
None	81 (57.40)
1	31 (22)
2	21 (14.90)
3	8 (5.70)
Total number of medication types usually taken	
None	84 (59.60)
1	28 (19.90)
2	19 (13.50)
3	8 (5.70)
4	2 (1.40)
Sensory neuropathy symptoms	0 (0.0)
Motor neuropathy symptoms	7 (4.96)
**End of the study**	
Line of therapy	
Adjuvant	103 (73)
Neoadjuvant	38 (27)
Regimen (mg/m^2^)	
Paclitaxel 175 mg/m^2^ given every 3 weeks	97 (68.80)
Paclitaxel 80 mg/m^2^ given every week	28 (19.90)
Docetaxel 80 mg/m^2^ given every 3 weeks	16 (11.30)
Number of cycles received	
2	10 (7.10)
3	9 (6.40)
4	122 (86.50)
Cumulative dose (mg), mean (SD)	1131.63 (268.75)
Sensory neuropathy symptoms	125 (89)
Motor neuropathy symptoms^a^	59 (44.03)

^a^ n = 134.

### The incidence density of TIPN symptoms

**Figure 1:**
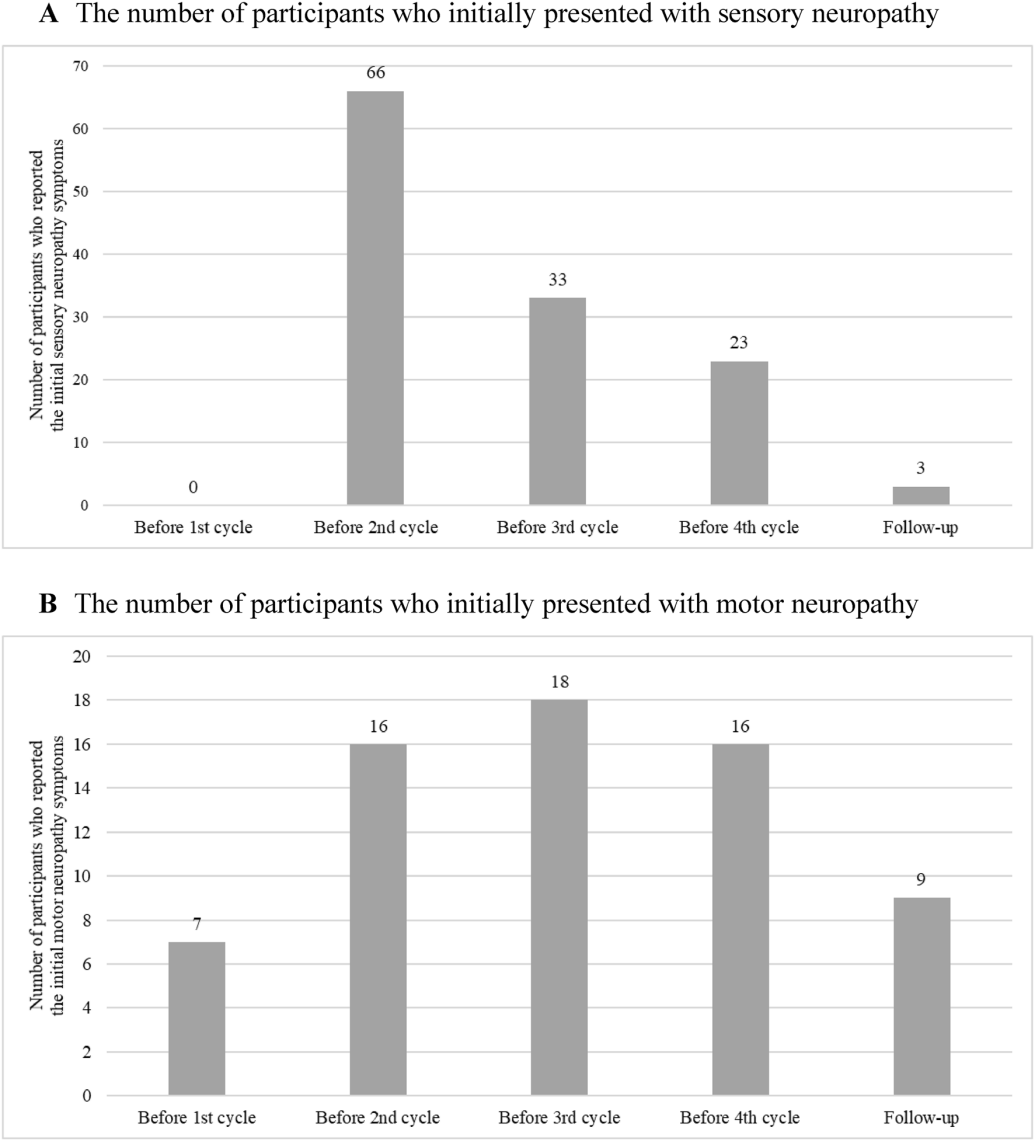
The number of participants who reported initial symptoms of (**A**) The sensory neuropathy symptoms, and (**B**) The motor neuropathy symptoms.

Over the course of treatment, sensory neuropathy was detected in 125 out of 141 participants (89%), 67 of whom were found to have sensory neuropathy alone, while the other 58 participants had both sensory and motor neuropathy. For motor neuropathy symptoms, seven participants reported signs of motor neuropathy not included in computing incidence density; thus, the total number of participants was 134. Motor neuropathy was detected in 59 out of 134 participants (44.03%), with one found to have motor neuropathy alone. The sensory neuropathy incidence density was 21 per 1000 person-days. On the other hand, the motor neuropathy incidence density was 6 per 1000 person-days (Table 2). Furthermore, the highest number of cases of initial sensory neuropathy were documented (66 cases) before beginning the second treatment cycle. Then, before receiving the third and fourth cycle and follow-up, the occurrences reduced gradually (33, 23, and 3 cases, respectively). The highest number of cases of initial motor neuropathy were reported (18 cases) before receiving the third cycle. Then, before receiving the fourth and follow-up cycles, the occurrences decreased gradually (16 and 9 cases, respectively; Fig. 1).

**Table 2:** Taxane-induced peripheral neuropathy (TIPN) incidence density (1000 person-day), (total n = 141).

TIPN type	TIPN symptoms	Person-day	Incidence density (1000 person-days)
Sensory peripheral neuropathy	125	5850	21
Motor peripheral neuropathy	59	9124	6

*TIPN* taxane-induced peripheral neuropathy.

### Risk factors associated with TIPN symptoms

The results demonstrated a significant link between age and the incidence density of sensory and motor neuropathy (HR = 1.02; 95% CI: 1.01–1.05, *p* = 0.02 and HR = 1.05; 95% CI: 1.01–1.08, *p* = 0.01; Table 3). Participants who were equal to or older than 60 years old had a higher incidence of sensory and motor neuropathy symptoms than participants who were younger than 60 years.

**Table 3:** Cox multivariate model for factors associated with TIPN symptoms.

	β ± SE	HR_unadj_	95% CI	*P*-value	β ± SE	HR_adj_	95% CI	*P*-value
**Sensory peripheral neuropathy**								
Age	0.26 ± 0.01	1.03	1.00–1.05	0.02*	0.03 ± 0.01	1.02	1.01–1.05	0.02*
BMI	−0.01 ± 0.01	0.99	0.98–1.01	0.70	–	–	–	–
Line of therapy	0.07 ± 0.20	1.07	0.72–1.59	0.73	–	–	–	–
Docetaxel 80 mg/m^2^ given every 3 weeks		–	–	0.37		–	–	–
Paclitaxel 175 mg/m^2^ given every 3 weeks	0.36 ± 0.30	1.41	0.79–2.54	0.25	–	–	–	–
Paclitaxel 80 mg/m^2^ given every week	0.48 ± 0.35	1.62	0.82–3.19	0.36	–	–	–	–
Number of cycles received	–0.29 ± 0.17	0.75	0.53–1.05	0.09	–0.33–0.17	0.72	0.51–1.01	0.06
Cumulative dose	0.00 ± 0.00	1.00	0.99–1.00	0.34	–	–	–	–
Hypertension	0.14 ± 0.20	1.14	0.78–1.69	0.49	–	–	–	–
Dyslipidemia	0.37 ± 0.23	1.44	0.92–2.26	0.11	–	–	–	–
Diabetes	–0.39 ± 0.29	0.67	0.38–1.20	0.18	–	–	–	–
Others	–0.20 ± 0.26	0.82	0.49–1.37	0.45	–	–	–	–
Number of medical comorbidities	0.10 ± 0.09	1.11	0.92–1.33	0.26	–	–	–	–
Number of tablets usually taken	0.08 ± 0.08	1.09	0.92–1.28	0.34	–	–	–	–
**Motor peripheral neuropathy**								
Age	−0.39 ± 0.18	0.85	0.63–1.06	0.09	0.05 ± 0.02	1.05	1.01–1.08	0.01*
BMI	0.01 ± 0.01	1.01	0.99–1.03	0.39	–	–	–	–
Line of therapy	0.23 ± 0.28	1.26	0.72–2.19	0.42	–	–	–	–
Docetaxel 80 mg/m^2^ given every 3 weeks		–	–	0.27	–	–	–	–
Paclitaxel 175 mg/m^2^ given every 3 weeks	−0.23 ± 0.41	0.80	0.35–1.79	0.58	–	–	–	–
Paclitaxel 80 mg/m^2^ given every week	0.25 ± 0.46	1.28	0.53–3.13	0.58	–	–	–	–
Number of cycles received	0.04 ± 0.33	1.04	0.54–1.99	0.90	–	–	–	–
Cumulative dose	0.00 ± 0.00	1.00	1.00–1.01	0.21	0.01 ± 0.01	1.01	1.00–1.07	0.07
Hypertension	0.40 ± 0.28	1.49	0.87–2.56	0.15	–	–	–	–
Dyslipidemia	−0.43 ± 0.32	0.65	0.35–1.21	0.18	–	–	–	–
Diabetes	0.62 ± 0.38	1.85	0.87–3.91	0.11	–	–	–	–
Other	−0.56 ± 0.33	0.57	0.30–1.08	0.09	–	–	–	–
Number of medical comorbidities	0.34 ± 0.13	1.50	1.11–1.81	0.03*	–	–	–	–
Number of tablets usually taken	0.23 ± 0.12	0.78	0.45–1.95	0.12	–	–	–	–

*TIPN* taxane-induced peripheral neuropathy.

*Statistically significant.

### The rate of participants without TIPN symptoms overall

**Figure 2:**
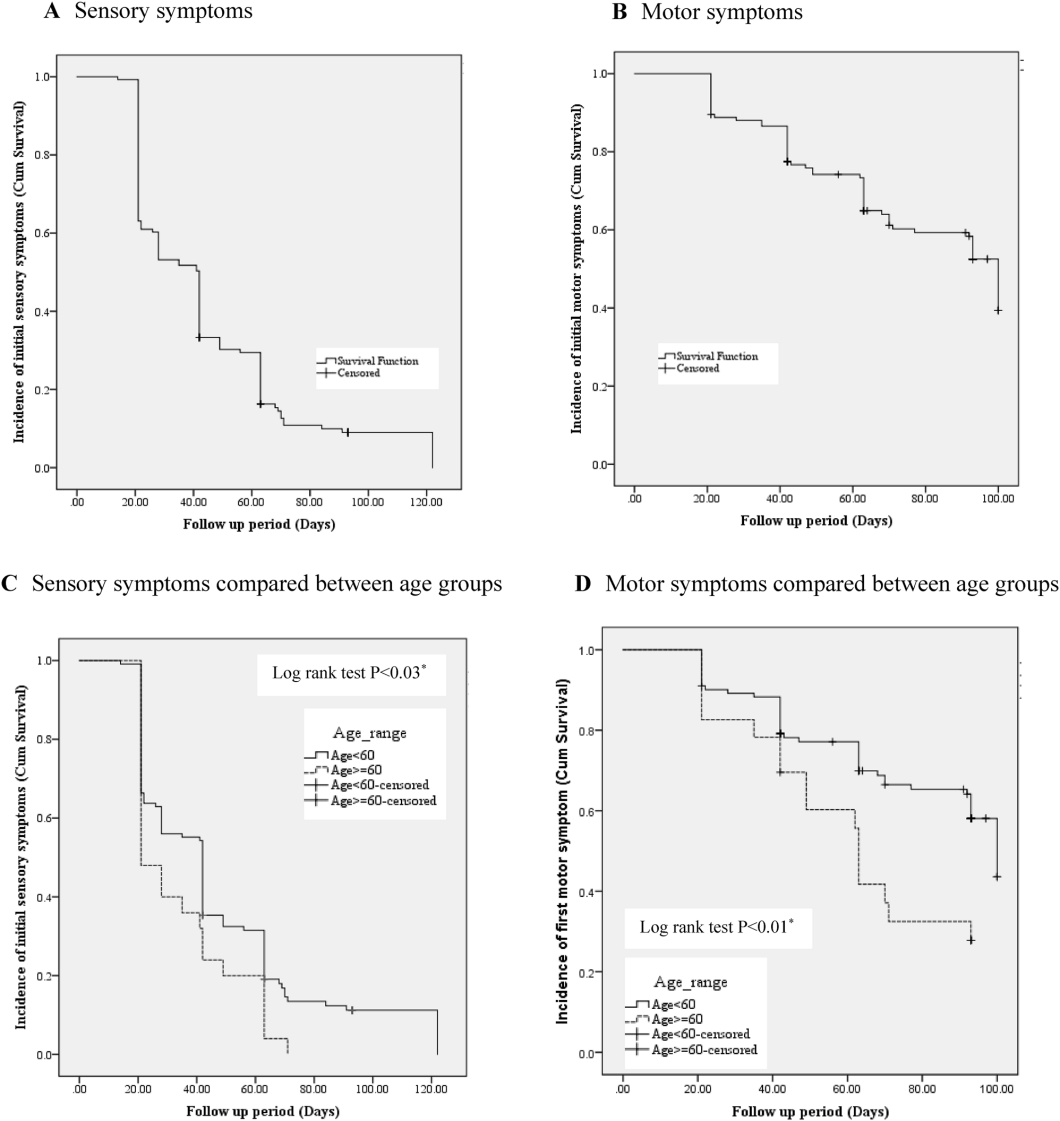
A Kaplan–Meier curve for the TIPN symptoms among women with breast cancer during received taxane-based chemotherapy. (**A**) Sensory neuropathy symptoms occurrence total women with breast cancer, (**B**) Motor neuropathy symptoms occurrence total women with breast cancer, (**C**) Sensory neuropathy symptoms occurrence between women with breast cancer who had equal or older than 60 years and women with breast cancer who had lower than 60 years, (**D**) Motor neuropathy symptoms occurrence between women with breast cancer who had equal or older than 60 years and women with breast cancer who had lower than 60 years.

Figure 2 illustrates the probability of initial TIPN symptom occurrence during the period of chemotherapy treatment among participants. Based on the survival analysis and KM curve, the overall rate of participants without sensory neuropathy symptoms was 11.3%. The median time without sensory neuropathy symptoms was 42 days after baseline (Fig. 2A), the overall rate of participants without motor neuropathy symptoms was 56%, and the median time without motor neuropathy symptoms was 100 days after baseline (Fig. 2B). Significantly different probabilities existed for sensory and motor neuropathy symptom occurrence between participants who were equal to or older than 60 years and participants aged under 60 years (*p* < 0.03 and *p* < 0.01; Fig. 2C and D).

### The severity of sensory and motor neuropathy

**Figure 3:**
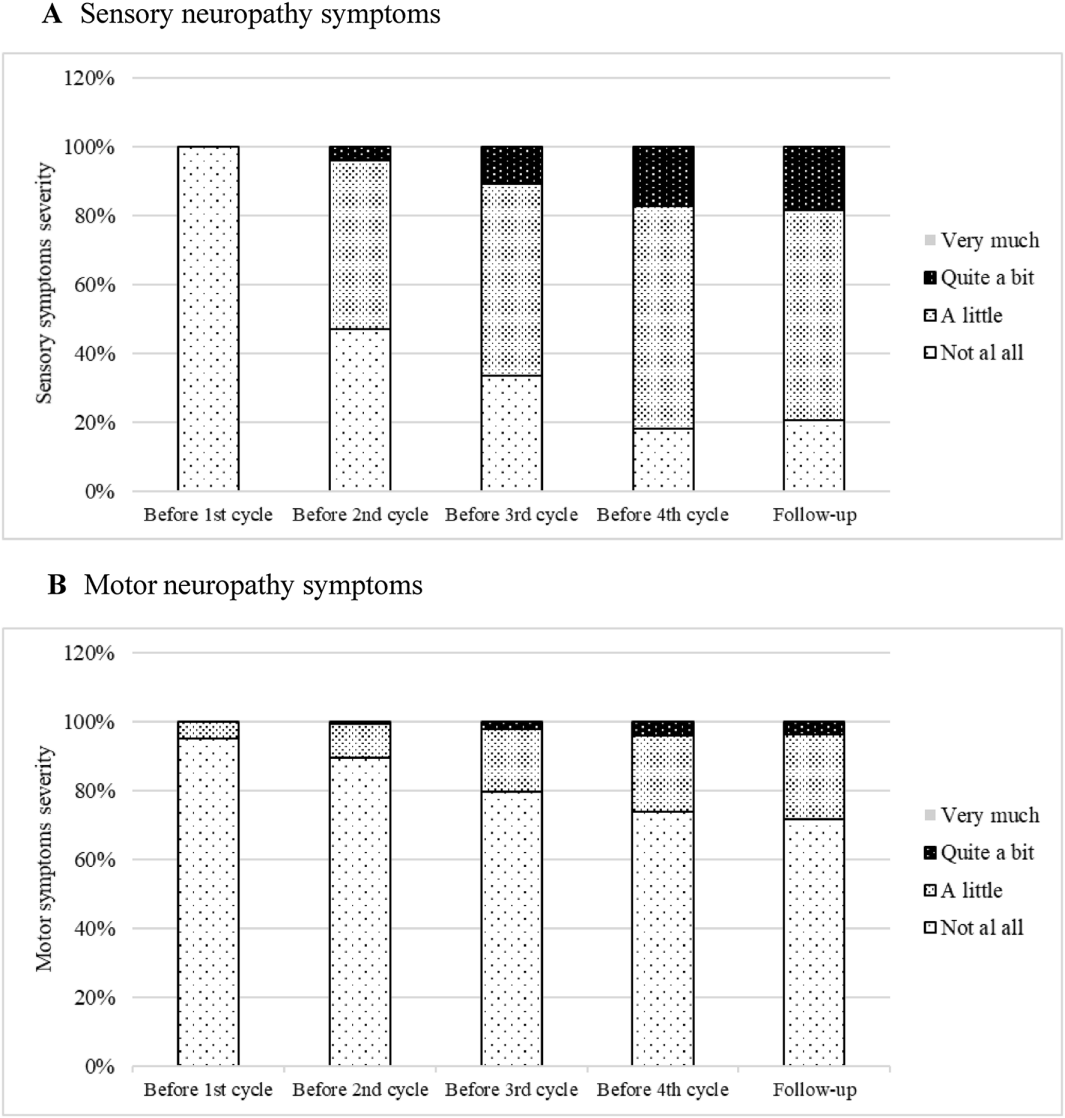
TIPN symptoms severity among participants during received taxane-based chemotherapy. (**A**) Sensory neuropathy, (**B**) Motor neuropathy.

The severity of TIPN symptoms was evaluated using the EORTC QLQ-CIPN20 questionnaire. Figure 3A and B demonstrate sensory and motor neuropathy. For sensory neuropathy, all participants (100%) reported “not at all” before receiving the first cycle. Severity was shown before receiving the second cycle then gradually increased before receiving the third cycle, the fourth cycle, and follow-up. For motor neuropathy, most participants (95%) reported “not at all” before receiving the first cycle, while 5% reported “a little”. Minor severity was shown before receiving the first cycle. After that, both minor severity and quite a bit gradually increased before receiving the second cycle, before receiving the third cycle, the fourth cycle, and follow-up.

## Discussion

This study examined the incidence density and investigated the possible risk factors associated with TIPN symptoms among women with breast cancer during taxane-based chemotherapy. The sensory neuropathy incidence density was 21 per 1000 person-days, and the motor neuropathy incidence density was 6 per 1000 person-days. In addition, the current study suggested that age was an important factor related to sensory and motor neuropathy.

This research was the first to determine the incidence density of sensory and motor neuropathy in women with breast cancer during taxane-based chemotherapy. The results showed that the incidence density of sensory neuropathy was higher than that of motor neuropathy over time. Moreover, the severity of sensory neuropathy symptoms seemed to deteriorate quickly over time when compared with motor neuropathy symptoms. The mechanism associated with the development of TIPN is complex and has not been fully understood. The literature suggests that several pathological factors are associated with TIPN symptoms, such as drugs affecting ion channels, mitochondrial dysfunction, axonal transport disorders, and inhibition of neuro-immune response^15^. Taxane treatment has generally been accumulated in the dorsal root ganglia (DRG) since the blood–brain barrier cannot be crossed. Furthermore, the majority of sensory neurons with cell bodies in DRG induce predominantly sensory neuropathy^18^. The possible cause of distal symmetrical sensory neuropathy among patients receiving neurotoxic agents might be associated with the mitotoxicity hypothesis^19^. The mitochondria of the sensory axon may be susceptible rather than the motor axon mitochondria to taxane-based chemotherapy^20^. The mitochondrial disorder would lead to a long-term energy deficit in axons that would lead to mostly sensory neuropathy^21^. According to the severity of TIPN symptoms, most of the participants indicated their degree of symptoms as “a little” and “quite a bit”, while no participants reported “very much”. The literature has indicated that taxane-based chemotherapy with weekly schedules seemed to increase the risk of TIPN symptoms compared to an every 3 weeks schedule^22,23^. Most participants (80%) in this study received taxane-based chemotherapy every 3 weeks, which might be associated with the low severity in patients.

However, the results differed from a previous study that suggested that motor neuropathy was slightly more frequently reported than sensory neuropathy^14^. Several studies have suggested that TIPN symptoms commonly present with predominantly sensory symptoms with less prominent motor involvement^1,3,24^. In general, individuals with motor neuropathy frequently have more severe neuropathy^9,25^. The disparity in results might be attributed to differences in follow-up time. The current study was conducted over a relatively short time (3-month follow-up), whereas the previous study was conducted over a longer period (12-month follow-up). Chronic neuropathy can persist for months or years after treatment cessation^1,26^. Another gap between the two studies might be related to the differences in the assessment tool seeming to affect the results. The previous study used a clinician-based scale known as the National Cancer Institute Common Toxicity Criteria (NCI-CTC), which appears to have several limitations in evaluating TIPN^27,28^. Although the NCI-CTC is widely recognized, considerable inter-observer disagreement exists when using this measure^29^. The previous study found that the EORTC QLQ-CIPN20 may be more likely to be accurate and sensitive than the NCI-CTC^28^. The EORTC QLQ-CIPN20 is a patient-reported questionnaire, which assesses the complication of chemotherapy treatment due to functional impairment. On the other hand, the NCI-CTC evaluates the complications of chemotherapy treatment based on a combination of impairment, disability, and QOL assessments, which may result in misinterpretation^27,30^. Therefore, TIPN symptoms should be assessed early to detect sensory neuropathy symptoms; those with motor neuropathy symptoms appear to have greater severity symptoms and should be aware of an increased risk of falls^9^.

Our results demonstrated a significant link between age and the incidence density of both sensory and motor neuropathy. These findings are consistent with prior studies that evaluated the risk factors associated with TIPN in patients with cancer who received taxane-based chemotherapy^4,8,26^, even though sensory and motor neuropathy were not separately identified in these investigations. The results indicated a 4% rise in the frequency of TIPN when patient age increased by 1 year^8^. Similarly, Bao et al.^4^ determined the prevalence and risk factors of TIPN symptoms in women with breast cancer who had undergone taxane-based chemotherapy. The results showed the association between older age and the prevalence of sensory neuropathy. Advanced age was also related to a higher risk and longer duration of TIPN symptoms^26^. The link between aging and TIPN symptoms might be explained by the possibility of mitotoxicity, with mitochondria discovered as important contributors causing TIPN^31^. In addition, aging mitochondria may be a deficit that increases the risk of TIPN in older people^32^. The peripheral nervous system structure is also relevant in older people because of the declining number of neurons and nerve fibers as well as the regenerative and reinnervating capacity of the nerve fibers^33^. From both hypotheses, age might be an important factor associated with TIPN symptoms. Accordingly, early screening for TIPN symptoms including sensory and motor neuropathy symptoms among women with breast cancer should be performed during taxane-based chemotherapy, particularly in older women.

Previous studies have reported several factors including the chemotherapy conditions^22,23,34^ and health and medical conditions^8^. However, the current study found that the incidence of TIPN symptoms was not associated with chemotherapy conditions or health and medical conditions. One of the possible reasons might be related to the small number of participants in the current study. In addition, the results differed from Hershman et al.^8^, who suggested that health and medical conditions were associated with TIPN symptoms. The disparity in results might be attributed to differences in the age of participants. The current study was conducted in younger women (35–65 years), whereas the previous study was conducted in older people (≥ 65 years). Comorbidities are more common in older than in younger persons^35^.

Several limitations must be considered. Only 35–65-year-old women with breast cancer received taxane-based treatment. Therefore, the current study’s findings may not apply to other cancer populations, chemotherapy agents, or age categories. Other limitations include the small sample size and the relatively short period of the investigation (3-month follow-up). Further studies with larger sample sizes and various types of cancer and chemotherapy agents over a longer period should be conducted.

In conclusion, the findings showed that women with breast cancer following taxane-based chemotherapy treatment had a higher incidence of sensory neuropathy than motor neuropathy. Furthermore, age was linked to sensory and motor neuropathy. These findings imply that screening may be necessary to detect early TIPN symptoms and provide appropriate rehabilitation programs, particularly for older people.

## Data Availability

The datasets analyzed in this study are not publicly available. Please contact the corresponding author regarding any reasonable requests for the data.

## Code availability

The statistical analysis code is available upon reasonable request.
